# *N*,*N*′-Bis(2-chloro­phenyl­sulfon­yl)suberamide

**DOI:** 10.1107/S1600536811009196

**Published:** 2011-03-15

**Authors:** Vinola Z. Rodrigues, Sabine Foro, B. Thimme Gowda

**Affiliations:** a Department of Chemistry, Mangalore University, Mangalagangotri 574 199, Mangalore, India; b Institute of Materials Science, Darmstadt University of Technology, Petersenstrasse 23, D-64287 Darmstadt, Germany

## Abstract

In the crystal of the title compound, C_20_H_22_Cl_2_N_2_O_6_S_2_, the asymmetric unit comprises half of a mol­ecule, the remaining portion is generated *via* an inversion centre. The conformation of the N—H and C=O bonds in the SO_2_–NH–C(O)–C segment is *anti*. The mol­ecule is bent at the S atom with the C–SO_2_–NH–C(O) torsion angle being 68.16 (19)°. The dihedral angle between the plane of the benzene ring and the SO_2_–NH–C(O)–C segment is 77.5 (1)°. Hydrogen bonds of the type N—H⋯O(C) link mol­ecules into supra­molecular chains along the *b* axis.

## Related literature

For the study of the effect of substituents on the structures of *N*-(ar­yl)-amides, see: Gowda *et al.* (2000 ▶). For the effect of substituents in *N*-(ar­yl)-aryl­sulfonamides, see: Gowda *et al.* (2005 ▶). For the effect of substituents on the structures of *N*-(aryl­sulfon­yl)-amides, see: Rodrigues *et al.* (2011 ▶).
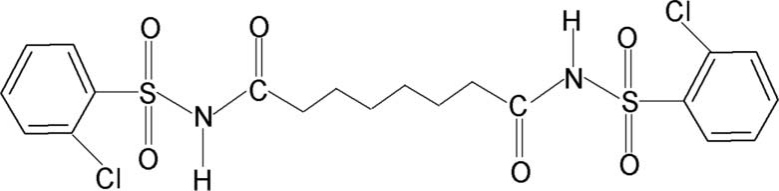


## Experimental

### 

#### Crystal data

C_20_H_22_Cl_2_N_2_O_6_S_2_
*M* *_r_* = 521.42
Monoclinic, 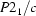

*a* = 7.8737 (9) Å
*b* = 9.717 (1) Å
*c* = 14.616 (2) Å
β = 94.575 (9)°
*V* = 1114.7 (2) Å^3^
*Z* = 2
Mo *K*α radiation
μ = 0.52 mm^−1^
*T* = 293 K
0.36 × 0.22 × 0.10 mm

#### Data collection

Oxford Diffraction Xcalibur diffractometer with a Sapphire CCD detector
Absorption correction: multi-scan (*CrysAlis RED*; Oxford Diffraction, 2009 ▶) *T*_min_ = 0.835, *T*_max_ = 0.950
4126 measured reflections
2276 independent reflections
1744 reflections with *I* > 2σ(*I*)
*R*_int_ = 0.018

#### Refinement

*R*[*F* ^2^ > 2σ(*F* ^2^)] = 0.038
*wR*(*F* ^2^) = 0.104
*S* = 1.05
2276 reflections
148 parameters
1 restraint
H atoms treated by a mixture of independent and constrained refinement
Δρ_max_ = 0.32 e Å^−3^
Δρ_min_ = −0.33 e Å^−3^

### 

Data collection: *CrysAlis CCD* (Oxford Diffraction, 2009 ▶); cell refinement: *CrysAlis RED* (Oxford Diffraction, 2009 ▶); data reduction: *CrysAlis RED*; program(s) used to solve structure: *SHELXS97* (Sheldrick, 2008 ▶); program(s) used to refine structure: *SHELXL97* (Sheldrick, 2008 ▶); molecular graphics: *PLATON* (Spek, 2009 ▶); software used to prepare material for publication: *SHELXL97*.

## Supplementary Material

Crystal structure: contains datablocks I, global. DOI: 10.1107/S1600536811009196/tk2727sup1.cif

Structure factors: contains datablocks I. DOI: 10.1107/S1600536811009196/tk2727Isup2.hkl

Additional supplementary materials:  crystallographic information; 3D view; checkCIF report

## Figures and Tables

**Table 1:** Hydrogen-bond geometry (Å, °)

*D*—H⋯*A*	*D*—H	H⋯*A*	*D*⋯*A*	*D*—H⋯*A*
N1—H1*N*⋯O3^i^	0.83 (2)	2.20 (2)	3.020 (2)	172 (2)

Symmetry code: (i) 
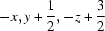
.

**Fig. 1.**
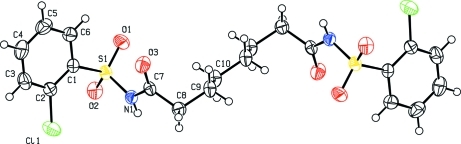
Molecular structure of (I), showing the atom labelling scheme and displacement ellipsoids at the 50% probability level.

**Fig. 2.**
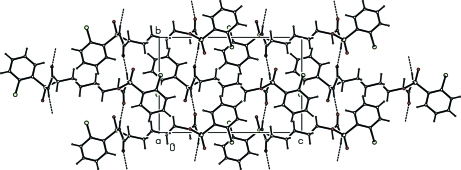
Molecular packing of (I) viewed in projection down the a axis and with hydrogen bonding shown as dashed lines.

## supplementary crystallographic information

### Comment

The amide and sulfonamide moieties are important constituents of many biologically significant compounds. As part of an investigation studying the effect of substituents on the structures of this class of compounds (Gowda *et al.*, 2000, 2005; Rodrigues *et al.*, 2011), in the present work, the structure of *N*,*N*-bis(2-chlorophenylsulfonyl)-suberamide (I) has been determined (Fig. 1). The asymmetric unit comprises half of a molecule, the remaining portion is generated through an inversion centre, similar to that observed in *N*,*N*-bis(2-chlorophenylsulfonyl)-adipamide (II) (Rodrigues *et al.*, 2011). The conformation of the N—H and C═O bonds in the SO_2_—NH—C(═O)—C segment is *anti*. The molecule is bent at the S atom with the C—SO_2_—NH—C(═O) torsion angle being 68.16 (19) °, compared to the value of -65.1 (6)° in (II). The torsion angles C2—C1—S1—N1 and C6—C1—S1—N1 are, respectively, 70.6 (2)° and -113.32 (17)°. The corresponding values in (II) are -69.5 (6) ° and 108.8 (5) °, respectively. The dihedral angle between the planes of the benzene ring and the SO_2_—NH—C(═O)—C segment in (I) is 77.5 (1) °, compared to the value of 89.6 (2) ° in (II).

A series of N—H···O(C) intermolecular hydrogen bonds (Table 1) link the molecules into chains running along the *b* axis (Fig. 2).

### Experimental

Compound (I) was prepared by refluxing a mixture of suberic acid (0.01 mol) with 2-chlorobenzenesulfonamide (0.02 mol) and POCl_3_ for 1 h on a water bath. The reaction mixture was allowed to cool and diethyl ether added. The solid product obtained was filtered, washed thoroughly with ether and hot ethanol. The compound was recrystallized to a constant melting point. Colourless prisms were grown by the slow evaporation of its ethanol solution at room temperature.

### Refinement

The H atom of the NH group was located in a difference map and later restrained to the distance N—H = 0.86±0.02 Å. The other H atoms were positioned with idealized geometry using a riding model with aromati-C—H distance = 0.93 Å, and methylene-C—H = 0.97 Å. All H atoms were refined with *U*_iso_(H) = 1.2*U*_eq_(N,C).

### Crystal data

**Table:**

C_20_H_22_Cl_2_N_2_O_6_S_2_	*F*(000) = 540
*M**_r_* = 521.42	*D*_x_ = 1.553 Mg m^−^^3^
Monoclinic, *P*2_1_/*c*	Mo *K*α radiation, λ = 0.71073 Å
Hall symbol: -P 2ybc	Cell parameters from 1476 reflections
*a* = 7.8737 (9) Å	θ = 2.6–27.7°
*b* = 9.717 (1) Å	µ = 0.52 mm^−^^1^
*c* = 14.616 (2) Å	*T* = 293 K
β = 94.575 (9)°	Prism, colourless
*V* = 1114.7 (2) Å^3^	0.36 × 0.22 × 0.10 mm
*Z* = 2	

### Data collection

**Table:**

Oxford Diffraction Xcalibur diffractometer with a Sapphire CCD detector	2276 independent reflections
Radiation source: fine-focus sealed tube	1744 reflections with *I* > 2σ(*I*)
graphite	*R*_int_ = 0.018
Rotation method data acquisition using ω scans	θ_max_ = 26.4°, θ_min_ = 2.6°
Absorption correction: multi-scan (*CrysAlis RED*; Oxford Diffraction, 2009)	*h* = −9→9
*T*_min_ = 0.835, *T*_max_ = 0.950	*k* = −11→12
4126 measured reflections	*l* = −18→8

### Refinement

**Table:**

Refinement on *F*^2^	Primary atom site location: structure-invariant direct methods
Least-squares matrix: full	Secondary atom site location: difference Fourier map
*R*[*F*^2^ > 2σ(*F*^2^)] = 0.038	Hydrogen site location: inferred from neighbouring sites
*wR*(*F*^2^) = 0.104	H atoms treated by a mixture of independent and constrained refinement
*S* = 1.05	*w* = 1/[σ^2^(*F*_o_^2^) + (0.0588*P*)^2^ + 0.1117*P*] where *P* = (*F*_o_^2^ + 2*F*_c_^2^)/3
2276 reflections	(Δ/σ)_max_ = 0.001
148 parameters	Δρ_max_ = 0.32 e Å^−^^3^
1 restraint	Δρ_min_ = −0.33 e Å^−^^3^

### Special details

**Table:**

Experimental. CrysAlis RED (Oxford Diffraction, 2009) Empirical absorption correction using spherical harmonics, implemented in SCALE3 ABSPACK scaling algorithm.
Geometry. All e.s.d.'s (except the e.s.d. in the dihedral angle between two l.s. planes) are estimated using the full covariance matrix. The cell e.s.d.'s are taken into account individually in the estimation of e.s.d.'s in distances, angles and torsion angles; correlations between e.s.d.'s in cell parameters are only used when they are defined by crystal symmetry. An approximate (isotropic) treatment of cell e.s.d.'s is used for estimating e.s.d.'s involving l.s. planes.
Refinement. Refinement of *F*^2^ against ALL reflections. The weighted *R*-factor *wR* and goodness of fit *S* are based on *F*^2^, conventional *R*-factors *R* are based on *F*, with *F* set to zero for negative *F*^2^. The threshold expression of *F*^2^ > σ(*F*^2^) is used only for calculating *R*-factors(gt) *etc*. and is not relevant to the choice of reflections for refinement. *R*-factors based on *F*^2^ are statistically about twice as large as those based on *F*, and *R*- factors based on ALL data will be even larger.

### Fractional atomic coordinates and isotropic or equivalent isotropic displacement parameters (Å^2^)

**Table:**

	*x*	*y*	*z*	*U*_iso_*/*U*_eq_	
C1	0.3294 (3)	−0.0856 (2)	0.61075 (13)	0.0301 (5)	
C2	0.2763 (3)	−0.0520 (2)	0.52035 (14)	0.0329 (5)	
C3	0.3042 (3)	−0.1437 (3)	0.45110 (15)	0.0449 (6)	
H3	0.2664	−0.1228	0.3908	0.054*	
C4	0.3873 (4)	−0.2654 (3)	0.47050 (17)	0.0499 (7)	
H4	0.4073	−0.3258	0.4232	0.060*	
C5	0.4412 (3)	−0.2986 (3)	0.55920 (18)	0.0479 (6)	
H5	0.4977	−0.3813	0.5719	0.057*	
C6	0.4118 (3)	−0.2096 (2)	0.62958 (16)	0.0381 (5)	
H6	0.4473	−0.2327	0.6898	0.046*	
C7	0.0104 (3)	−0.0734 (2)	0.75061 (14)	0.0306 (5)	
C8	−0.1521 (3)	−0.0352 (2)	0.79001 (14)	0.0347 (5)	
H8A	−0.2460	−0.0863	0.7594	0.042*	
H8B	−0.1743	0.0622	0.7808	0.042*	
C9	−0.1371 (3)	−0.0685 (2)	0.89303 (14)	0.0385 (6)	
H9A	−0.2477	−0.0554	0.9167	0.046*	
H9B	−0.1066	−0.1647	0.9011	0.046*	
C10	−0.0071 (3)	0.0185 (3)	0.94906 (14)	0.0394 (6)	
H10A	−0.0384	0.1147	0.9422	0.047*	
H10B	0.1034	0.0065	0.9252	0.047*	
N1	0.1046 (2)	0.03505 (19)	0.72033 (12)	0.0316 (4)	
H1N	0.066 (3)	0.1134 (18)	0.7259 (16)	0.038*	
O1	0.3959 (2)	−0.03761 (18)	0.78343 (10)	0.0456 (4)	
O2	0.3555 (2)	0.16371 (17)	0.68236 (10)	0.0415 (4)	
O3	0.0630 (2)	−0.19135 (16)	0.74794 (11)	0.0437 (4)	
Cl1	0.17624 (9)	0.10214 (7)	0.49044 (4)	0.0505 (2)	
S1	0.31012 (7)	0.02626 (6)	0.70503 (3)	0.03155 (17)	

### Atomic displacement parameters (Å^2^)

**Table:**

	*U*^11^	*U*^22^	*U*^33^	*U*^12^	*U*^13^	*U*^23^
C1	0.0299 (11)	0.0338 (12)	0.0268 (10)	−0.0019 (9)	0.0051 (8)	−0.0017 (9)
C2	0.0336 (12)	0.0347 (12)	0.0305 (11)	−0.0013 (10)	0.0026 (9)	0.0015 (9)
C3	0.0513 (15)	0.0557 (16)	0.0275 (11)	−0.0037 (13)	0.0025 (11)	−0.0034 (11)
C4	0.0576 (17)	0.0482 (16)	0.0459 (14)	−0.0019 (13)	0.0167 (12)	−0.0152 (12)
C5	0.0488 (16)	0.0374 (14)	0.0586 (16)	0.0075 (11)	0.0113 (13)	−0.0039 (12)
C6	0.0377 (13)	0.0377 (13)	0.0390 (12)	0.0018 (10)	0.0038 (10)	0.0024 (10)
C7	0.0389 (12)	0.0300 (12)	0.0232 (9)	−0.0026 (10)	0.0040 (9)	−0.0009 (9)
C8	0.0356 (12)	0.0334 (12)	0.0353 (12)	−0.0025 (10)	0.0045 (9)	0.0021 (10)
C9	0.0441 (14)	0.0399 (14)	0.0329 (12)	−0.0059 (11)	0.0123 (10)	−0.0002 (10)
C10	0.0453 (14)	0.0409 (14)	0.0335 (12)	−0.0062 (11)	0.0125 (10)	−0.0010 (10)
N1	0.0384 (11)	0.0264 (10)	0.0310 (9)	0.0014 (8)	0.0092 (8)	0.0012 (8)
O1	0.0493 (11)	0.0561 (11)	0.0298 (8)	0.0067 (8)	−0.0074 (7)	−0.0001 (8)
O2	0.0447 (10)	0.0362 (9)	0.0440 (9)	−0.0111 (7)	0.0068 (7)	−0.0030 (7)
O3	0.0529 (11)	0.0277 (9)	0.0527 (10)	0.0006 (8)	0.0187 (8)	−0.0006 (7)
Cl1	0.0632 (4)	0.0445 (4)	0.0418 (3)	0.0062 (3)	−0.0077 (3)	0.0065 (3)
S1	0.0340 (3)	0.0352 (3)	0.0253 (3)	−0.0016 (2)	0.0016 (2)	−0.0017 (2)

### Geometric parameters (Å, °)

**Table:**

C1—C6	1.385 (3)	C7—C8	1.492 (3)
C1—C2	1.393 (3)	C8—C9	1.535 (3)
C1—S1	1.771 (2)	C8—H8A	0.9700
C2—C3	1.379 (3)	C8—H8B	0.9700
C2—Cl1	1.732 (2)	C9—C10	1.516 (3)
C3—C4	1.370 (4)	C9—H9A	0.9700
C3—H3	0.9300	C9—H9B	0.9700
C4—C5	1.370 (4)	C10—C10^i^	1.527 (4)
C4—H4	0.9300	C10—H10A	0.9700
C5—C6	1.378 (3)	C10—H10B	0.9700
C5—H5	0.9300	N1—S1	1.6532 (19)
C6—H6	0.9300	N1—H1N	0.826 (16)
C7—O3	1.221 (3)	O1—S1	1.4250 (16)
C7—N1	1.381 (3)	O2—S1	1.4282 (17)
			
C6—C1—C2	119.5 (2)	C7—C8—H8B	109.9
C6—C1—S1	116.55 (16)	C9—C8—H8B	109.9
C2—C1—S1	123.79 (17)	H8A—C8—H8B	108.3
C3—C2—C1	119.4 (2)	C10—C9—C8	114.08 (18)
C3—C2—Cl1	118.01 (18)	C10—C9—H9A	108.7
C1—C2—Cl1	122.59 (17)	C8—C9—H9A	108.7
C4—C3—C2	120.5 (2)	C10—C9—H9B	108.7
C4—C3—H3	119.8	C8—C9—H9B	108.7
C2—C3—H3	119.8	H9A—C9—H9B	107.6
C5—C4—C3	120.4 (2)	C9—C10—C10^i^	112.9 (2)
C5—C4—H4	119.8	C9—C10—H10A	109.0
C3—C4—H4	119.8	C10^i^—C10—H10A	109.0
C4—C5—C6	120.1 (2)	C9—C10—H10B	109.0
C4—C5—H5	120.0	C10^i^—C10—H10B	109.0
C6—C5—H5	120.0	H10A—C10—H10B	107.8
C5—C6—C1	120.1 (2)	C7—N1—S1	124.07 (16)
C5—C6—H6	119.9	C7—N1—H1N	117.4 (17)
C1—C6—H6	119.9	S1—N1—H1N	115.6 (17)
O3—C7—N1	120.9 (2)	O1—S1—O2	118.86 (10)
O3—C7—C8	123.3 (2)	O1—S1—N1	108.65 (10)
N1—C7—C8	115.66 (19)	O2—S1—N1	104.39 (10)
C7—C8—C9	108.97 (18)	O1—S1—C1	107.08 (11)
C7—C8—H8A	109.9	O2—S1—C1	110.79 (10)
C9—C8—H8A	109.9	N1—S1—C1	106.41 (10)
			
C6—C1—C2—C3	1.0 (3)	C7—C8—C9—C10	66.2 (3)
S1—C1—C2—C3	176.93 (18)	C8—C9—C10—C10^i^	−179.1 (2)
C6—C1—C2—Cl1	−178.69 (17)	O3—C7—N1—S1	−17.2 (3)
S1—C1—C2—Cl1	−2.8 (3)	C8—C7—N1—S1	160.00 (15)
C1—C2—C3—C4	−1.7 (4)	C7—N1—S1—O1	−46.8 (2)
Cl1—C2—C3—C4	178.1 (2)	C7—N1—S1—O2	−174.61 (16)
C2—C3—C4—C5	1.1 (4)	C7—N1—S1—C1	68.16 (19)
C3—C4—C5—C6	0.1 (4)	C6—C1—S1—O1	2.7 (2)
C4—C5—C6—C1	−0.7 (4)	C2—C1—S1—O1	−173.29 (18)
C2—C1—C6—C5	0.2 (3)	C6—C1—S1—O2	133.79 (17)
S1—C1—C6—C5	−176.03 (19)	C2—C1—S1—O2	−42.2 (2)
O3—C7—C8—C9	64.1 (3)	C6—C1—S1—N1	−113.32 (17)
N1—C7—C8—C9	−113.0 (2)	C2—C1—S1—N1	70.6 (2)

Symmetry codes: (i) −*x*, −*y*, −*z*+2.

### Hydrogen-bond geometry (Å, °)

**Table:**

*D*—H···*A*	*D*—H	H···*A*	*D*···*A*	*D*—H···*A*
N1—H1N···O3^ii^	0.83 (2)	2.20 (2)	3.020 (2)	172 (2)

Symmetry codes: (ii) −*x*, *y*+1/2, −*z*+3/2.

## References

[bb1] Gowda, B. T., Paulus, H. & Fuess, H. (2000). *Z. Naturforsch. Teil A*, **55**, 791–800.

[bb2] Gowda, B. T., Shetty, M. & Jayalakshmi, K. L. (2005). *Z. Naturforsch. Teil A*, **60**, 106–112.

[bb3] Oxford Diffraction (2009). *CrysAlis CCD* and *CrysAlis RED* Oxford Diffraction Ltd, Yarnton, Oxfordshire, England.

[bb4] Rodrigues, V. Z., Foro, S. & Gowda, B. T. (2011). *Acta Cryst.* E**67**, o837. 10.1107/S1600536811008464 PMC3100076 21754120

[bb5] Sheldrick, G. M. (2008). *Acta Cryst.* A**64**, 112–122. 10.1107/S0108767307043930 18156677

[bb6] Spek, A. L. (2009). *Acta Cryst.* D**65**, 148–155. 10.1107/S090744490804362X PMC2631630 19171970

